# Prolonged Ileus Due to Underlying Shigella Infection After Bilateral Open Inguinal Hernia Repair

**DOI:** 10.7759/cureus.55474

**Published:** 2024-03-04

**Authors:** Claire Dalby, Michelle Lippincott, Jarrod Olafson, Paul C Kuo

**Affiliations:** 1 Surgery, University of South Florida Morsani College of Medicine, Tampa, USA

**Keywords:** general surgery complication, infectious disease, shigella, umbilical hernia repair, inguinal hernia repair, ileus

## Abstract

We present a rare case of prolonged ileus caused by underlying *Shigella* infection after surgical hernia repair. Infectious disease is an uncommon cause of postoperative prolonged ileus in adults.

Our 48-year-old male patient underwent bilateral open inguinal hernia repair and open umbilical hernia repair without complication at an academic institution, with same-day discharge. Eight days later, he presented to the emergency department with complaints of severe cramping abdominal pain, nausea, emesis, and watery diarrhea. Physical examination, computed tomography scan of the abdomen and pelvis, and abdominal X-ray were initially concerning for bowel obstruction. The patient was admitted to the general surgery service. Concern for ileus with underlying gastritis arose after a small bowel follow-through showed contrast eventually reaching the rectum. A subsequent gastrointestinal pathogens panel was positive for *Shigella*. The patient’s symptoms resolved after appropriate antibiotic treatment. Shigellosis and other infectious diseases should be considered in the differential diagnosis of postoperative prolonged ileus.

## Introduction

This case report presents a rare case of prolonged ileus caused by underlying *Shigella* infection (shigellosis) after bilateral open inguinal hernia repair and open umbilical hernia repair in an adult patient at an academic institution. Infectious causes of prolonged ileus are uncommon, particularly in adulthood. Only one study has described intestinal obstruction due to *Shigella*, and a majority of the study population consisted of pediatric patients [1]. While a few case reports have described intestinal obstruction caused by parasites, and, more rarely, bacteria, none have described ileus with underlying shigellosis. To our knowledge, this is the first report of prolonged ileus caused by shigellosis after inguinal hernia repair in an adult.

## Case presentation

Our patient was a 48-year-old male who initially presented to the general surgery clinic with a complaint of right groin swelling. Past medical history included lumbar degenerative disc disease, ulnar nerve palsy, and umbilical hernia. Past surgical history was significant for left hand surgery. Current medications included diclofenac 50 mg three times daily as needed for pain. His family history was significant for arthritis in his mother and diabetes and asthma in his father. He had a 0.5-pack-year smoking history and had quit smoking 10 years prior. He used marijuana socially and did not drink alcohol. He was afebrile with stable vital signs. Physical examination was notable for bilateral inguinal hernias, right greater than left, and an umbilical hernia.

The patient was scheduled for bilateral open inguinal hernia repair with mesh and open umbilical hernia repair. This approach was chosen due to patient preference. The standard approach in the senior author’s practice is laparoscopic totally extraperitoneal, followed by laparoscopic transabdominal preperitoneal (TAPP), or robotic TAPP when robot time is available. The open approach is reserved for scrotal hernias, selected patients with ascites, or patient preferences. Repair occurred without complication and the patient was discharged the same day. Eight days later, the patient was brought to the emergency department by Emergency Medical Services for complaints of one week of worsening, now severe, cramping abdominal pain, nausea, emesis, intolerance of intake by mouth, and non-bloody watery diarrhea. General surgery was consulted due to concern for intestinal obstruction.

**Figure 1:**
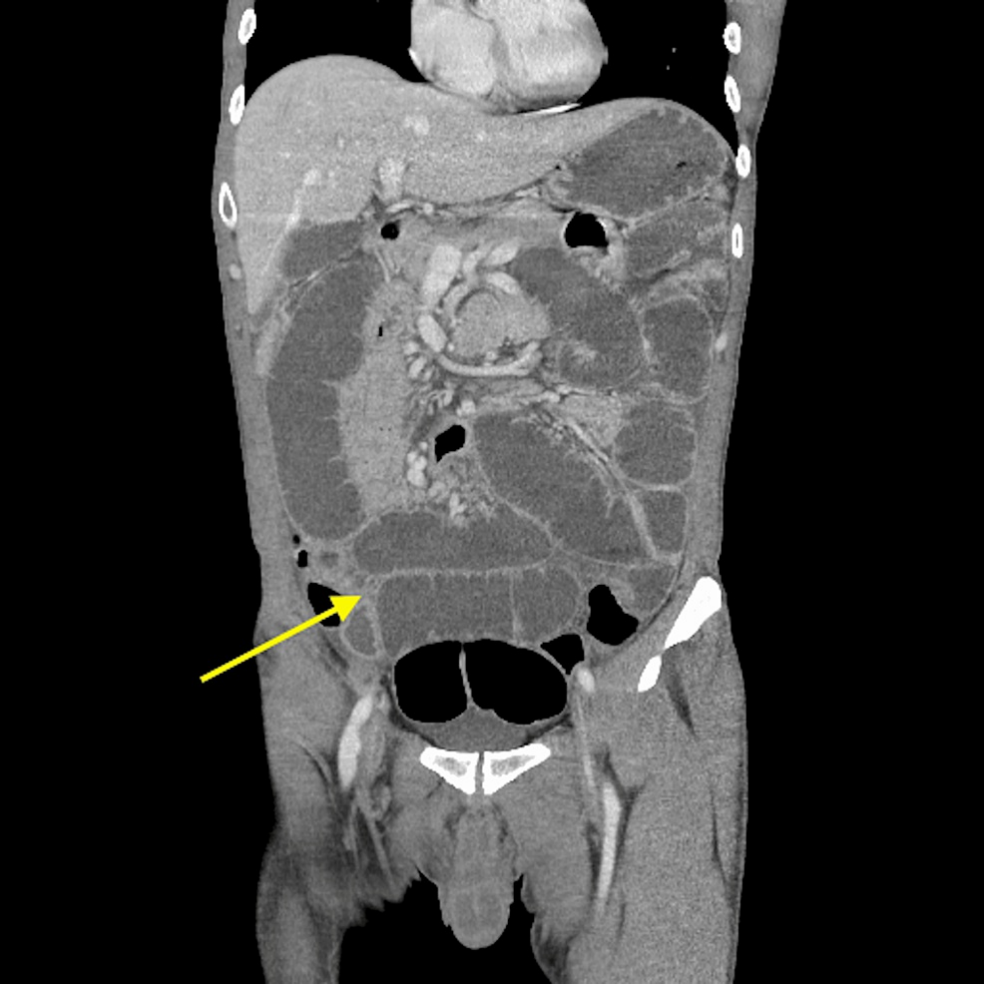
Computerized tomography of the abdomen and pelvis with intravenous contrast, coronal view. The image depicts distended loops of the proximal small bowel with a probable transition point in the right hemiabdomen-right lower quadrant.

**Figure 2:**
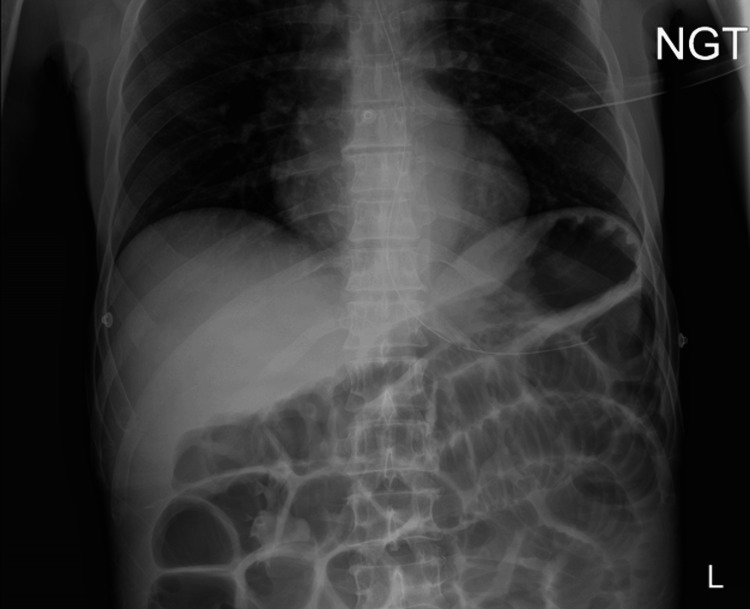
X-ray of the abdomen. The image depicts diffuse elevated loops of the small bowel with a nasogastric tube (NGT) in place.

Physical examination revealed a moderately distended and tympanic abdomen, diffusely tender to palpation, particularly in the epigastric region, with mild voluntary guarding but no peritonitis. Bilateral groin incisions were clean, dry, and intact, with no hernia recurrence appreciated. Laboratory testing was significant for an elevated white blood cell count of 11.28 × 10^3^/mL, hyponatremia of 130 mmol/L, hyperkalemia of 5.6 mmol/L, elevated aspartate transaminase of 76 U/L, and low serum albumin of 2.9 g/dL. Computerized tomography (CT) scan of the abdomen and pelvis with intravenous (IV) contrast revealed findings initially concerning for an acute high-grade small bowel obstruction (SBO). The scan showed a markedly distended stomach and loops of proximal small bowel with internal air-fluid levels with a probable transition point in the right hemiabdomen-right lower quadrant, without pneumatosis intestinalis or portal venous gas (Figure 1). However, the CT scan demonstrated gas and fluid distal to the transition point, an unusual finding in high-grade bowel obstruction. Abdominal X-ray revealed diffuse elevated loops of the small bowel concerning for SBO (Figure 2).

The patient was admitted to general surgery for the management of possible SBO. Small bowel follow-through on day three of inpatient care revealed delayed transit of contrast through the small bowel, with terminal ileum and cecum visualized after nine hours. Nine hours is considered a prolonged small bowel follow-through, which raises concerns for prolonged ileus. Due to increasing concern for prolonged ileus caused by underlying gastritis, a BIOFIRE FilmArray gastrointestinal (GI) pathogens panel by bioMérieux was ordered on day four of inpatient care. This panel was positive for* Shigella*/enteroinvasive *Escherichia coli* (EIEC).

Given the above findings, including a distended abdomen and a CT scan with a distended stomach and small bowel with a probable transition point in the right lower quadrant, the surgical team was initially concerned about high-grade SBO. Additional differential diagnoses included reactive ileus and partial SBO. However, watery diarrhea is an uncommon symptom of postoperative SBO. Therefore, once the small bowel follow-through revealed that contrast had delayed transit through the small bowel but did eventually arrive at the rectum, the team began to consider prolonged ileus with an underlying infection. Diagnosis of shigellosis was confirmed by the detection of *Shigella* on the GI pathogens panel.

Once the GI pathogens panel was positive for *Shigella*/EIEC, the patient received pharmacologic treatment with ciprofloxacin 500 mg by mouth twice daily. Treatment was initiated on day four of inpatient care and continued until discharge on day eight. The patient’s bowel function returned without nausea or vomiting, and laboratory tests were within normal limits by the day of discharge. The patient did not return for follow-up.

## Discussion

This complication of prolonged ileus caused by underlying shigellosis after bilateral open inguinal hernia repair and open umbilical hernia repair, treated pharmacologically, represents a Grade 2 surgical complication by Clavien-Dindo Classification [2].

Infection with the gram-negative bacteria *Shigella* can cause shigellosis, which may manifest in a variety of GI symptoms, including bloody or non-bloody diarrhea, vomiting, and diffuse abdominal pain [3]. It may rarely cause intestinal obstruction [1]. While this infection is more common in developing countries, there are approximately 450,000 cases in the United States each year [3]. Shigellosis is more common in young children, but the disease can affect individuals of any age [3]. Spread of *Shigella* occurs mainly by fecal-oral contact, and these bacteria are highly contagious due to their resistance to stomach acid [3,4].

Prolonged ileus or intestinal obstruction due to infectious causes, such as shigellosis, is rare, particularly in adults. Only a single study has reported cases of intestinal obstruction due to *Shigella* infection, and a majority of this study population consisted of pediatric patients [1]. This study found the incidence of intestinal obstruction in 1,211 patients with shigellosis to be 2.5% [1]. Various case reports have described intestinal obstruction caused by parasites [5-12], *Chlamydia* *trachomatis* [13-15], intrauterine device infection [16], mpox [17], and *Mycobacterium avium *complex infection [18]. To our knowledge, this is the first case report of prolonged ileus caused by shigellosis after bilateral open inguinal hernia repair and open umbilical hernia repair in an adult.

Diagnosing *Shigella* as a cause of ileus presents a clinical challenge because bacterial infection, such as shigellosis, is an uncommon cause of prolonged ileus in adult patients. In addition, it is scarcely described in the literature. These challenges make it difficult to recognize and diagnose ileus due to infectious causes.

## Conclusions

Infectious causes of prolonged ileus and intestinal obstruction are scarcely described in the literature. Additionally, bacterial infection is a rare cause of prolonged ileus in adults. Therefore, underlying bacterial infection may not be routinely considered during the management of postoperative prolonged ileus. This patient’s underlying shigellosis could have been more rapidly identified and treated by the initial inclusion of underlying infection in the differential diagnosis and earlier obtainment of the GI pathogens panel. Shigellosis and other infectious diseases should be considered in the differential diagnosis of prolonged ileus after operative hernia repair.
